# Supplementary material for: Novel Driver Strength Index highlights important cancer genes in TCGA PanCanAtlas patients
Source: PeerJ. 2022 Aug 11;10:e13860. doi: 10.7717/peerj.13860 (PMC9375969; doi:10.7717/peerj.13860)
Supplement: Supplemental Information 2 [file peerj-10-13860-s002.zip › COHORTS/genes plots/cohorts/2021_8_16_14_9_distribution_cohorts_males_LUSC.pdf]

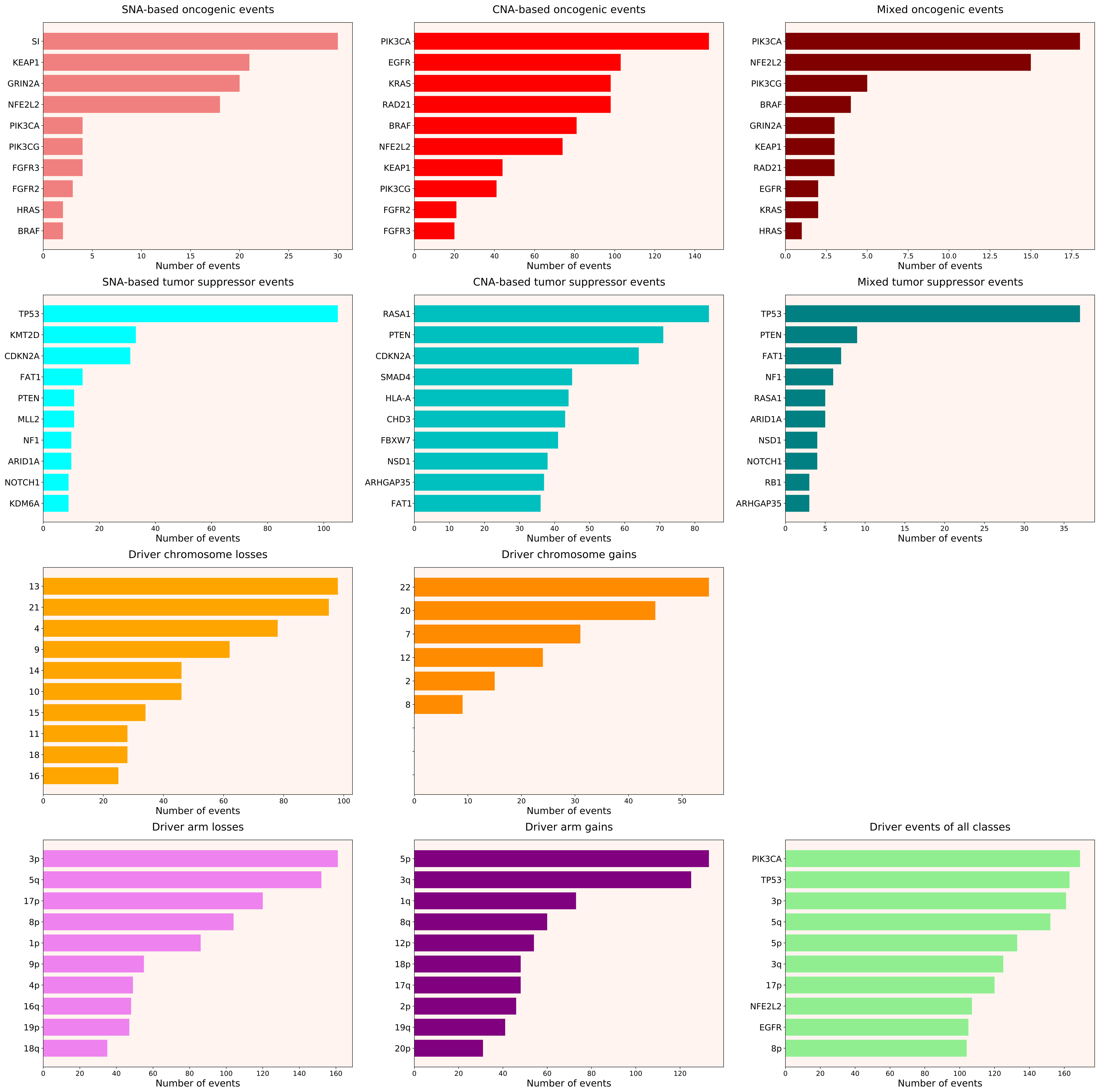

SNA-based oncogenic events
SI
KEAP1
GRIN2A
NFE2L2
PIK3CA
PIK3CG
FGFR3
FGFR2
HRAS
BRAF
0 5 10 15 20 25 30
Number of events
CNA-based oncogenic events
PIK3CA
EGFR
KRAS
RAD21
BRAF
NFE2L2
KEAP1
PIK3CG
FGFR2
FGFR3
0 20 40 60 80 100 120 140
Number of events
Mixed oncogenic events
PIK3CA
NFE2L2
PIK3CG
BRAF
GRIN2A
KEAP1
RAD21
EGFR
KRAS
HRAS
0.0 2.5 5.0 7.5 10.0 12.5 15.0 17.5
Number of events
SNA-based tumor suppressor events
TP53
KMT2D
CDKN2A
FAT1
PTEN
MLL2
NF1
ARID1A
NOTCH1
KDM6A
0 20 40 60 80 100
Number of events
CNA-based tumor suppressor events
RASA1
PTEN
CDKN2A
SMAD4
HLA-A
CHD3
FBXW7
NSD1
ARHGAP35
FAT1
0 10 20 30 40 50 60 70 80
Number of events
Mixed tumor suppressor events
TP53
PTEN
FAT1
NF1
RASA1
ARID1A
NSD1
NOTCH1
RB1
ARHGAP35
0 5 10 15 20 25 30 35
Number of events
Driver chromosome losses
13
21
4
9
14
10
15
11
18
16
0 20 40 60 80 100
Number of events
Driver chromosome gains
22
20
7
12
2
8
0 10 20 30 40 50
Number of events
Driver arm losses
3p
5q
17p
8p
1p
9p
4p
16q
19p
18q
0 20 40 60 80 100 120 140 160
Number of events
Driver arm gains
5p
3q
1q
8q
12p
18p
17q
2p
19q
20p
0 20 40 60 80 100 120
Number of events
Driver events of all classes
PIK3CA
TP53
3p
5q
5p
3q
17p
NFE2L2
EGFR
8p
0 20 40 60 80 100 120 140 160
Number of events
